# Atypical Presentation of Pulmonary Carcinoid Tumor With Pleural Involvement: Diagnostic and Surgical Challenges

**DOI:** 10.7759/cureus.87822

**Published:** 2025-07-13

**Authors:** Ayushi Sen, Ahmad Bahay, Shagun Thakur, Salma Gonzalez, Michael Zemaitis, Loren J Harris

**Affiliations:** 1 Surgery, American University of Antigua, St. John's, ATG; 2 Trauma Surgery, Richmond University Medical Center, Staten Island, USA; 3 Surgery, Richmond University Medical Center, Staten Island, USA

**Keywords:** 68ga-dotatate pet/ct, curative surgery, frozen section, lung cancer staging, m1a staging, neuroendocrine tumor, pleural involvement, pulmonary carcinoid tumor, somatostatin receptor imaging, typical carcinoid

## Abstract

This case report presents a diagnostically challenging pulmonary typical carcinoid tumor with localized pleural involvement, initially misdiagnosed intraoperatively as small cell carcinoma. Despite the presence of pleural nodules, typically staged as M1a disease, histopathology confirmed a low-grade neuroendocrine tumor with favorable features, including a low Ki-67 index and absence of necrosis. Somatostatin receptor imaging using 68Ga-DOTATATE positron emission tomography/computed tomography (PET/CT) revealed no distant spread. The patient underwent definitive surgical management with lobectomy, pleurectomy, and lymph node dissection. Postoperative recovery was uneventful, and the patient remains disease-free. This case highlights the limitations of frozen section diagnosis in neuroendocrine tumors and challenges the prognostic implications of pleural involvement in typical carcinoid tumors. Individualized surgical decisions based on tumor biology, rather than rigid staging criteria, may be warranted.

## Introduction

Pulmonary carcinoid tumors are rare neuroendocrine neoplasms that account for approximately 1-2% of all primary lung malignancies [1]. These tumors are classified into two categories: typical and atypical carcinoids. Typical carcinoid tumors are well-differentiated, slow-growing neoplasms with an excellent prognosis when diagnosed early and treated surgically [1-2]. Typical carcinoid tumors are not commonly associated with aggressive behavior, such as pleural invasion or extensive lymphatic spread. The presence of pleural nodules in a typical carcinoid tumor is an exceedingly rare finding that poses significant diagnostic and therapeutic challenges, as it may mimic metastatic disease.

## Case presentation

**Figure 1:**
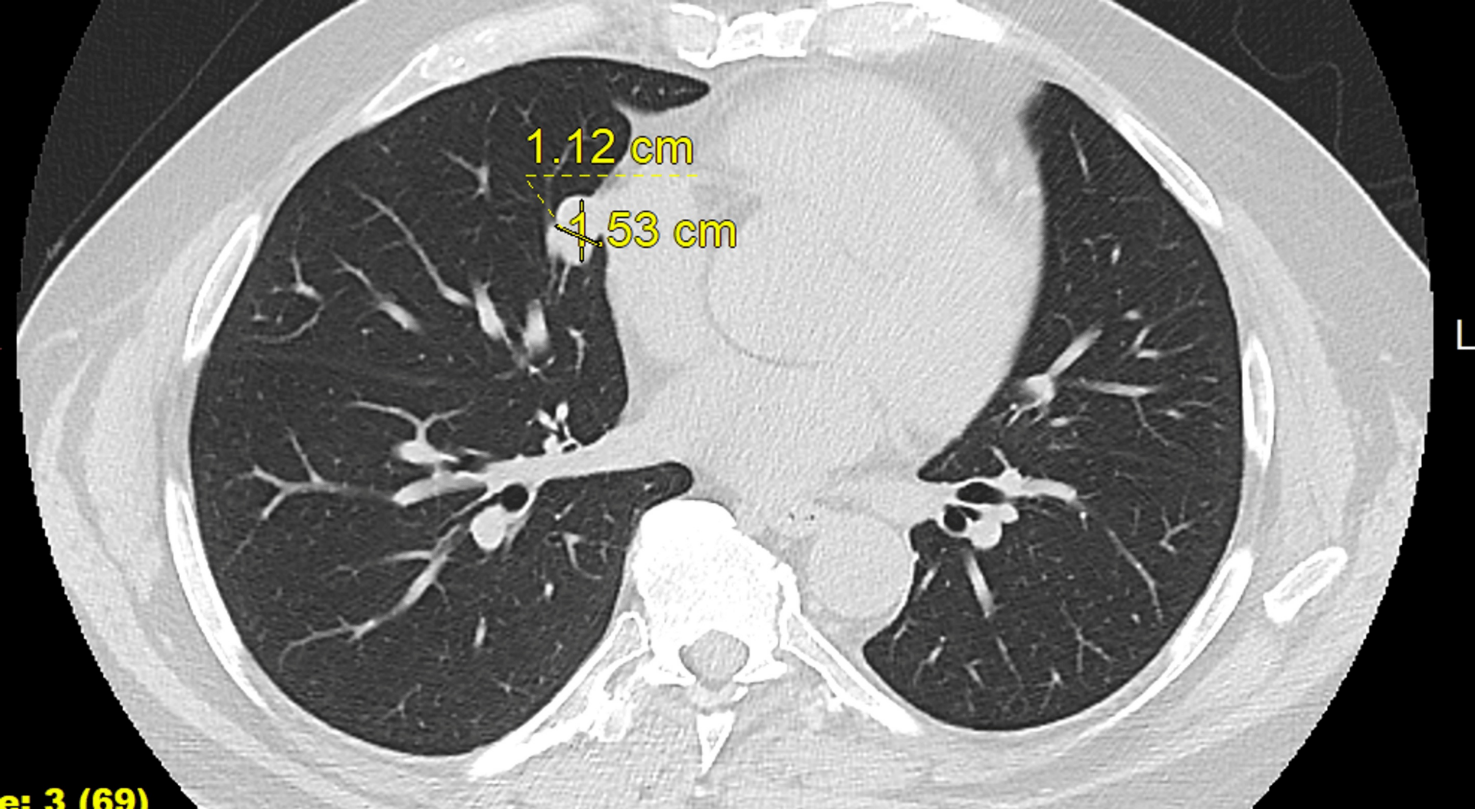
Chest CT of a 1.5 cm right middle lobe pulmonary nodule

A 58-year-old male with no respiratory symptoms underwent coronary calcium scoring in March 2024 as part of a routine cardiovascular risk assessment. The imaging incidentally revealed a 1.5 cm solitary pulmonary nodule located in the right middle lobe. The patient had no history of smoking and no significant pulmonary or systemic symptoms. A follow-up computed tomography (CT) scan (Figure 1), one month after the initial imaging, confirmed the presence of the nodule, which remained stable in size over time. Positron emission tomography/computed tomography (PET/CT) imaging showed moderate fluorodeoxyglucose (FDG) uptake, with a maximum standardized uptake value (SUV) of 3.4. This intermediate uptake raised suspicion for a low-grade malignancy, including the possibility of a carcinoid tumor [3].

A CT-guided transthoracic needle biopsy was performed, and histopathological evaluation identified a spindle cell neoplasm consistent with a typical carcinoid tumor. Immunohistochemical staining demonstrated positivity for chromogranin and synaptophysin, and a quantified Ki-67 proliferative index of approximately 1%. These findings supported the diagnosis of a well-differentiated, low-grade neuroendocrine tumor. The patient was scheduled for a robotic-assisted right middle lobectomy with mediastinal lymph node dissection.

During the thoracoscopic surgical exploration, multiple small nodules were observed on the right parietal pleura. This finding was unexpected, as there was no evidence of pleural involvement on preoperative imaging. Intraoperative frozen section analysis of the pleural nodules yielded a preliminary diagnosis of small cell carcinoma, a high-grade neuroendocrine tumor with a vastly different prognosis and treatment approach. Given this unexpected diagnosis, the surgical team decided to abort the lobectomy and instead performed pleural biopsies to obtain additional tissue for definitive pathological assessment.

The patient recovered uneventfully from the initial diagnostic procedure. Final immunohistochemical examination of the pleural nodules contradicted the frozen section results, revealing that the nodules were composed of typical carcinoid tumor cells identical in morphology and immunohistochemistry to the primary pulmonary lesion. There was no evidence of necrosis or increased mitotic activity, and the Ki-67 index remained low. To further assess the extent of disease and rule out distant metastasis, a 68Ga-DOTATATE PET/CT scan was performed, which did not reveal any extrathoracic disease.

**Figure 2:**
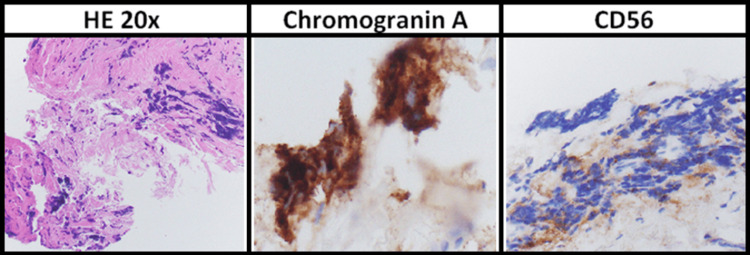
Typical carcinoid tumor hematoxylin and eosin staining at 20× showing positive chromogranin A and CD56

Given the confirmed diagnosis of a localized typical carcinoid tumor with pleural involvement, the patient underwent a definitive surgical resection, including a robotic-assisted right middle lobectomy, mediastinal lymph node dissection, and partial pleurectomy. Final pathological analysis (Figure 2) of the resected specimen confirmed a 1.8 cm typical carcinoid tumor with involvement of the visceral pleura. One subcarinal lymph node at station 7 tested positive for metastatic disease, classifying the tumor as pT2a pN2 pM0. Final pathology confirmed that only visceral pleural involvement was present, with no histologic evidence of parietal pleural invasion or pleural fluid positivity. According to American Joint Committee on Cancer (AJCC) 8th Edition criteria, this supports the M0 designation, as pleural nodules limited to the visceral surface do not constitute distant metastatic disease (M1a) [4]. The surgical margins were negative, and the Ki-67 index remained unchanged, confirming the low proliferative nature of the tumor. The patient recovered well postoperatively and was discharged without complications. He was followed closely for four months after surgery. A 68Ga-DOTATATE PET/CT performed in November 2024 showed no evidence of residual or extrathoracic disease. A follow-up chest CT in January 2025 confirmed no signs of recurrence or disease progression.

## Discussion

Pulmonary carcinoid tumors are uncommon neoplasms with favorable outcomes when managed surgically. Typical carcinoid tumors rarely metastasize or invade adjacent structures and are generally staged and treated as localized disease. This case, however, challenges these expectations due to the presence of multiple pleural nodules discovered intraoperatively and an initial frozen section diagnosis of small cell carcinoma, which significantly altered the operative course.

Pleural involvement by typical carcinoid tumors is exceptionally rare, with fewer than five well-documented cases in the literature. In general, lung cancer staging, parietal pleural involvement is classified as M1a disease, indicating distant spread and a poorer prognosis [5]. However, in the setting of low-grade carcinoid, this staging may not hold the same prognostic weight. The clinical dilemma arises: does pleural involvement in typical carcinoid truly signify metastatic spread, or is it an atypical but localized extension with curative potential?

This case supports the latter interpretation. Despite pleural involvement and pN2 nodal disease, the tumor demonstrated low proliferative activity, no necrosis, and histologically indolent behavior, aligning with what is expected of a typical carcinoid tumor. It is also important to note that small or flat pleural lesions, especially in low-grade tumors, are often below the resolution threshold of CT scans. This explains the discrepancy between the lack of pleural findings on preoperative imaging and the intraoperative discovery of nodules.

The misidentification of the pleural nodules as small cell carcinoma during intraoperative frozen section led to an aborted lobectomy. This underscores a significant challenge: neuroendocrine tumors often share overlapping cytologic features, and frozen section analysis can be misleading due to sampling limitations or crush artifact [6]. In neuroendocrine pathology, definitive diagnosis often hinges on formalin-fixed, paraffin-embedded tissue and immunohistochemistry, tools not available during frozen evaluation. This case illustrates the importance of cautious intraoperative interpretation when frozen sections yield unexpected or aggressive diagnoses that conflict with preoperative findings.

The PET/CT showed a moderate SUV (3.4) - not definitively benign or malignant. Such intermediate values are often associated with low-grade malignancies or even inflammatory conditions. While PET/CT is helpful, it lacks specificity, especially in neuroendocrine tumors. 68Ga-DOTATATE PET/CT was later utilized and showed no evidence of systemic spread, reaffirming the limited extent of disease. This suggests that somatostatin receptor-based imaging may be more appropriate in staging well-differentiated neuroendocrine tumors [3].

Following confirmation of a low-grade tumor, the patient underwent definitive resection, including a pleurectomy. While pleural involvement may technically meet M1a criteria, this case-and others like it-suggest that curative surgery can still be appropriate in selected patients with typical carcinoid tumors and localized pleural nodules [2-7].

The patient had no postoperative complications and remains disease-free to date. Long-term follow-up will be necessary to monitor for recurrence or progression, but the current trajectory is favorable.

## Conclusions

This case highlights an unusual and diagnostically challenging presentation of a pulmonary typical carcinoid tumor with pleural involvement. While pleural nodules are traditionally staged as M1a disease, often indicating distant metastasis and a worse prognosis, this classification may not be applicable to well-differentiated neuroendocrine tumors. In our patient, the absence of necrosis, low Ki-67 index, and indolent histologic features suggested a localized rather than metastatic behavior. The favorable biology and absence of distant disease supported a curative surgical approach, despite the presence of pleural and N2 nodal involvement. This underscores the importance of considering tumor behavior and histology alongside staging criteria in treatment decisions.

The case also illustrates the limitations of frozen section diagnosis in neuroendocrine tumors. The initial intraoperative misdiagnosis of small cell carcinoma, a high-grade malignancy, delayed definitive treatment, and altered the surgical plan. Given the cytologic overlap among neuroendocrine tumors and the potential for artifact in frozen sections, clinicians should interpret such results with caution, particularly when inconsistent with preoperative findings. Ultimately, individualized staging, careful use of somatostatin receptor imaging, and an adaptable surgical approach may offer the best outcomes for patients with atypical carcinoid presentations.
